# Vimentin Regulates Alternative Polyadenylation and mTOR Signaling via ARVCF to Promote B Cell Lymphoma Progression

**DOI:** 10.1155/humu/1463685

**Published:** 2025-09-28

**Authors:** Lujing Shao, Qianke Xing, Yao Xiong, Kaidi Jin, Qi Li, Chunyan Dong, Qianling Ye

**Affiliations:** ^1^Department of Oncology, East Hospital Affiliated to Tongji University, Tongji University School of Medicine, Tongji University, Shanghai, China; ^2^Tongji Hospital Affiliated to Tongji University, Tongji University School of Medicine, Tongji University, Shanghai, China; ^3^Department of Forensic Medicine, School of Basic Medical Sciences, Fudan University, Shanghai, China

**Keywords:** alternative polyadenylation, ARVCF, B cell lymphoma, mTOR signaling, vimentin

## Abstract

**Background:** Vimentin (VIM), a cytoskeletal protein implicated in tumor progression, has been associated with poor prognosis in B cell lymphomas. Alternative polyadenylation (APA), a posttranscriptional mechanism that modulates mRNA isoforms via 3⁣′UTR length changes, is frequently dysregulated in cancer. The interaction between VIM and APA in B cell lymphoma remains poorly understood.

**Methods:** We performed RNA-seq, APA analysis (DaPars), and proteomic profiling in wild-type and VIM-knockout (VIM-KO) B cell lymphoma cells (A20 and M12). Functional assays including CCK-8, EdU, Western blot, and ARVCF overexpression were used to explore the regulatory axis involving APA and mTOR signaling.

**Results:** VIM deletion in B cell lymphoma cells triggered widespread transcriptome remodeling, inducing 4089 APA shortening events that preferentially targeted prosurvival pathways, for example, mTORC1, G2-M checkpoint. Strikingly, RRAGA—a critical mTOR activator—underwent 3⁣′UTR shortening, concomitant with ARVCF downregulation in proteomic profiles. Functional rescue experiments demonstrated ARVCF's dual role in maintaining RRAGA 3⁣′UTR length and suppressing mTOR-EIF4G1 signaling, ultimately inhibiting lymphoma proliferation.

**Conclusion:** This study reveals a novel VIM–ARVCF–RRAGA–mTOR axis in B cell lymphoma, linking cytoskeletal disruption to APA-mediated oncogenic signaling. VIM loss drives APA shortening and mTOR activation via ARVCF downregulation, promoting lymphoma progression. These findings offer mechanistic insight and potential targets for therapeutic intervention.

## 1. Introduction

B cell lymphomas comprise a diverse group of malignancies arising from clonal expansions of mature B lymphocytes, accounting for approximately 85%–95% of lymphoid cancers [1, 2]. Common subtypes, such as diffuse large B cell lymphoma (DLBCL), follicular lymphoma (FL), and Burkitt lymphoma (BL), vary significantly in clinical behavior, prognosis, and genetic alterations [1, 3]. Despite therapeutic advances, the molecular mechanisms underlying B cell lymphomas remain incompletely understood, highlighting the need for deeper investigation to facilitate targeted therapies.

Vimentin (VIM), a Type III intermediate filament protein, critically regulates cellular architecture, signaling pathways, and invasive behavior of tumor cells [4–6]. In DLBCL, elevated VIM expression is associated with multidrug resistance and enhanced invasive capacity, implicating its involvement in aggressive disease phenotypes and poor clinical outcomes [7]. Additionally, VIM acts as a shared autoantigen recognized by lymphoma B cell receptors, further suggesting its potential role in tumor immunobiology and progression in B cell malignancies [8].

Alternative polyadenylation (APA) is a posttranscriptional regulatory mechanism generating multiple mRNA isoforms through differential cleavage and polyadenylation at distinct polyadenylation sites, predominantly in the 3⁣′ untranslated regions (3⁣′UTRs). APA is frequently dysregulated in cancer, resulting in shortened 3⁣′UTRs of oncogenes, which typically enhances mRNA stability by removing microRNA binding sites and regulatory elements, thus promoting oncogenic protein production and tumorigenesis [9]. In B cell malignancies, altered APA patterns can critically impact oncogene expression, exemplified by the RNA-binding protein NUDT21 regulating CD19 mRNA APA, which modulates B cell acute lymphoblastic leukemia sensitivity to CD19-targeted therapies [10]. Additionally, genome-wide analyses of acute lymphoblastic leukemia have revealed significant APA-associated differences in transcript isoforms among distinct leukemia subtypes, underscoring its potential as a subtype-specific regulatory mechanism in B cell malignancies [11].

Recent studies indicate that VIM may influence RNA processing by regulating RNA-binding proteins involved in APA [12]. VIM deficiency has been linked to altered expression of APA regulators, suggesting it may shape APA profiles indirectly. However, the role of VIM in modulating APA—and its impact on gene regulation—remains unexplored in the context of B cell lymphomas.

Therefore, this study is aimed at investigating how VIM influences APA regulation within the context of B cell lymphoma, specifically examining APA dynamics and the downstream molecular consequences resulting from VIM deficiency. Clarifying the interaction between VIM and APA will offer insights into novel regulatory mechanisms involved in B cell lymphoma biology and could highlight potential targets for therapeutic intervention.

## 2. Results

### 2.1. VIM Deletion Reprograms the Transcriptional Landscape Toward Prosurvival States in B Cell Lymphoma

**Figure 1:**
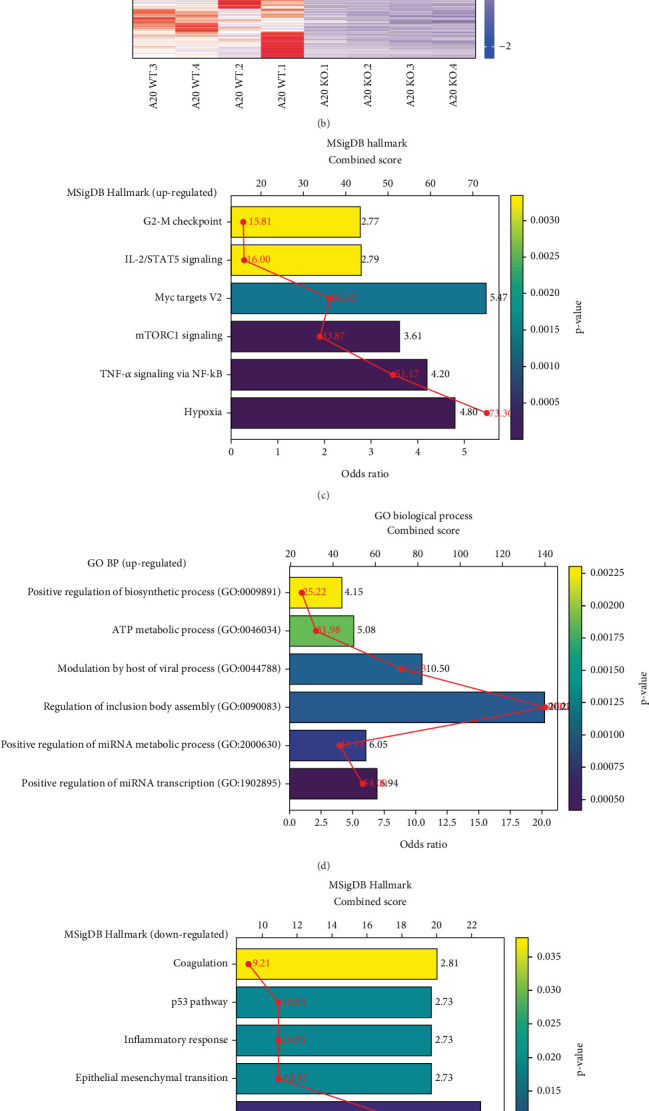
Vimentin knockout activates survival signaling in B cell lymphoma. (a) Volcano plot showing differentially expressed genes (DEGs) between wild-type (WT) and vimentin knockout (VIM-KO) B cell lymphoma. (b) Heatmap of significantly altered DEGs. (c) MSigDB Hallmark pathway enrichment analysis of upregulated DEGs. (d) GO enrichment analysis of upregulated DEGs. (e) MSigDB Hallmark pathway enrichment analysis of downregulated DEGs. (f) GO enrichment analysis of downregulated DEGs.

To investigate the role of VIM in B cell lymphoma, we performed RNA sequencing (RNA-seq) on wild-type (WT) and VIM-knockout (KO) B cell lymphoma cells. Differential expression analysis using DESeq2 revealed 686 significantly differentially expressed genes (DEGs), with 418 genes upregulated and 268 genes downregulated in VIM-KO B cell lymphoma cells (Figure 1a,b). The upregulated DEGs were enriched in MSigDB Hallmark terms related to cancer cell survival and proliferation, including hypoxia, TNF-alpha signaling via NF-*κ*B, mTORC1 signaling, IL-2/STAT5 signaling, and G2-M checkpoint (Figure 1c). Gene Ontology (GO) analysis further highlighted enrichment of the upregulated DEGs in processes such as positive regulation of miRNA transcription, regulation of inclusion body assembly, ATP metabolic process, and positive regulation of biosynthetic process (Figure 1d). In contrast, the downregulated DEGs were primarily associated with KRAS signaling up, apoptosis, epithelial mesenchymal transition, and inflammatory response (Figure 1e). GO analysis revealed that the downregulated DEGs were particularly enriched in immune-related processes, including antigen processing and presentation of exogenous peptide antigen, negative regulation of lymphocyte migration, positive regulation of B cell activation, and negative regulation of cytokine production (Figure 1f).

### 2.2. APA-Driven Transcript Shortening as a Mechanism of mTOR Activation in VIM-Deficient B Cell Lymphoma

**Figure 2:**
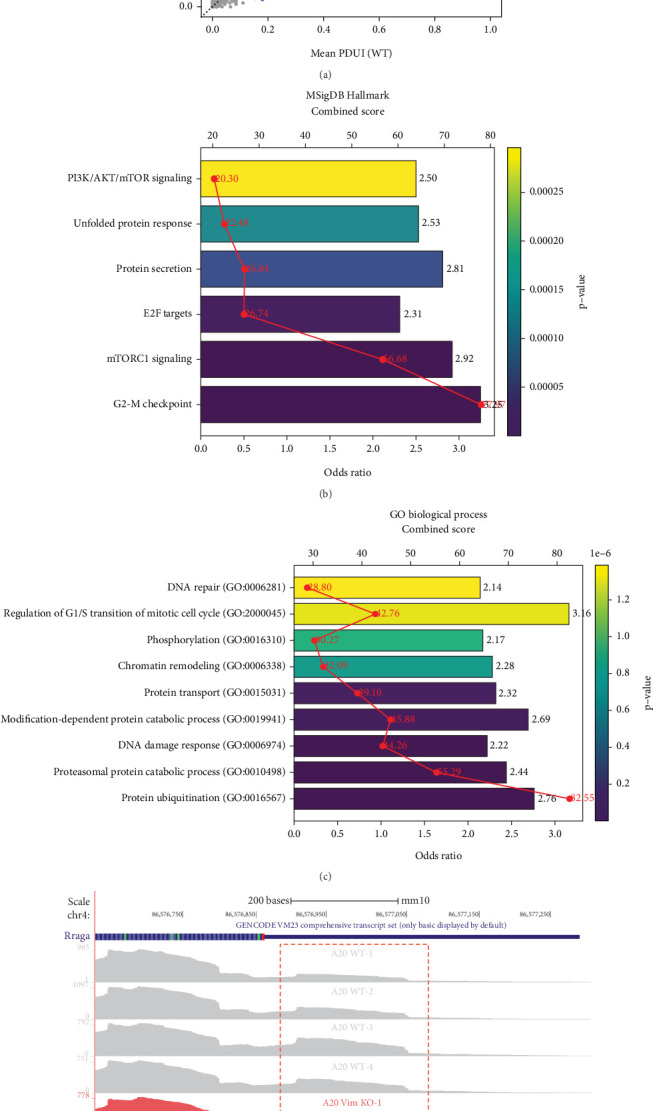
Vimentin knockout induces 3⁣′UTR shortening linked to survival signaling in B cell lymphoma. (a) Scatter plot of DaPars PDUI values comparing WT and VIM-KO cells, showing widespread 3⁣′UTR shortening in VIM-KO. (b) MSigDB Hallmark enrichment of shortened genes, indicating activation of survival-related pathways. (c) GO enrichment analysis of shortened genes, highlighting functions related to proliferation and survival. (d) RNA-seq track plot showing reduced 3⁣′UTR length of the RRAGA gene in VIM-KO cells.

APA is a posttranscriptional regulatory mechanism frequently dysregulated in cancer, though its role in VIM-regulated B cell lymphoma has not been well studied. Using the DaPars tool on our RNA-seq data, we observed widespread 3⁣′UTR shortening in VIM-KO B cell lymphoma cells, with 186 significant lengthening events and 4089 shortening events (Figure 2a). The shortened APA events were enriched in pathways such as G2-M checkpoint, mTORC1 signaling, E2F targets, protein secretion, and unfolded protein response (Figure 2b). GO analysis further revealed that these shortened genes were enriched in processes such as protein ubiquitination, proteasomal protein catabolic process, DNA damage response, modification-dependent protein catabolic process, chromatin remodeling, and regulation of G1/S transition of mitotic cell cycle (Figure 2c). Notably, one of the most significant shortening APA events was observed in the 3⁣′UTR of the **RRAGA** gene, which encodes RagA, a key protein involved in the activation of the mTOR pathway (Figure 2d). Previous studies have demonstrated that VIM-KO promotes cancer cell proliferation by activating the mTOR pathway [13]. Consistent with these findings, our results suggest that VIM-KO may drive mTOR activation in B cell lymphoma through APA-mediated regulation.

**Figure 3:**
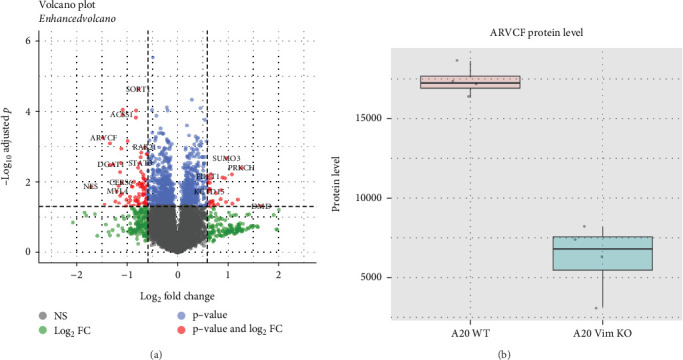
Proteomic profiling of VIM-KO B cell lymphoma. (a) Volcano plot showing differentially expressed proteins between WT and VIM-KO B cell lymphoma. (b) Boxplot illustrating reduced expression of ARVCF protein in VIM-KO cells.

To further elucidate the role of VIM in B cell lymphoma, we performed mass spectrometry on WT and VIM-KO B cell lymphoma cells. We identified 101 downregulated proteins and 32 upregulated proteins (Figure 3a). Although these differentially regulated proteins were not significantly enriched in specific pathways or GO terms, we noted that the splicing regulator **ARVCF** was among the top two downregulated proteins in VIM-KO B cell lymphoma cells (Figure 3b). Given that ARVCF has been reported to interact with hnRNPH proteins, which are crucial for APA regulation, this indicates that the massive gene shortening events observed in VIM-KO cells may be linked to the significant downregulation of ARVCF [14].

### 2.3. VIM-KO Promotes Lymphoma Proliferation via the mTOR-EIF4G1 Signaling Pathway

**Figure 4:**
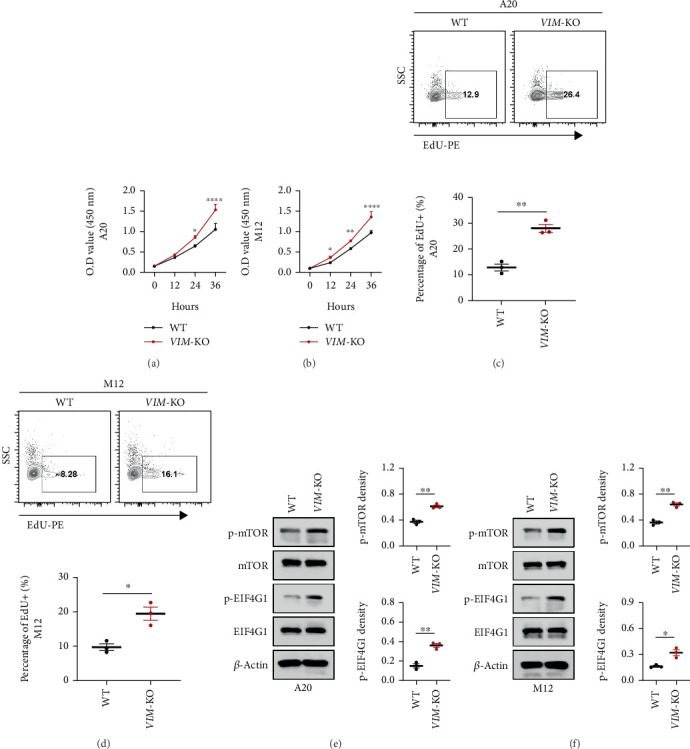
VIM knockout enhances lymphoma cell activity by activating the mTOR-EIF4G1 signaling pathway. (a, b) VIM-KO increases the viability of lymphoma cell lines. (c, d) VIM-KO promotes the proliferative capacity of lymphoma cell lines. (e, f) VIM-KO enhances the activity of the mTOR-EIF4G1 signaling pathway in lymphoma cells.

To determine whether VIM-KO influences the behavior of lymphoma cells, we compared the viability of WT lymphoma cell lines (A20 and M12) with that of VIM-KO variants (Figure 4a,b). CCK-8 assays revealed that VIM-KO significantly enhanced the viability of both lymphoma cell lines. EdU staining further confirmed the increased proliferative capacity of VIM-KO cells (Figure 4c,d).

Having established that VIM-KO enhances the proliferative activity of A20 and M12 cells, we explored the molecular mechanisms underlying this phenomenon. Previous studies have shown that the mTOR signaling pathway regulates tumor behavior by influencing the cell cycle, with the mTOR-EIF4G1 axis closely associated with tumor cell activity. We examined the activation status of the mTOR-EIF4G1 pathway in VIM-KO A20 and M12 cells. VIM-KO significantly increased the phosphorylation levels of mTOR and its downstream effector EIF4G1, indicating upregulation of the mTOR-EIF4G1 pathway (Figure 4e,f). Based on these findings, we propose that VIM-KO promotes lymphoma progression in vitro by enhancing the activity of the mTOR-EIF4G1 signaling pathway.

### 2.4. VIM-KO Enhances Lymphoma Cell Viability by Inhibiting Expression of the APA-Related Protein ARVCF

**Figure 5:**
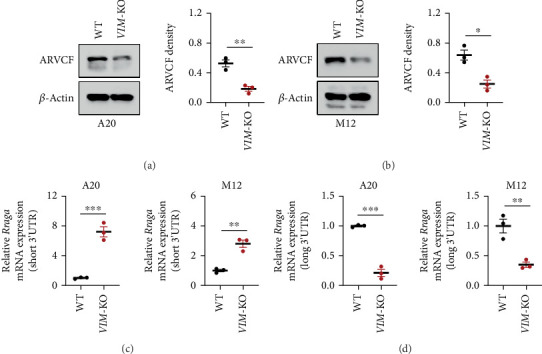
VIM knockout suppresses the expression of the APA-related protein ARVCF in lymphoma cell lines. (a, b) VIM-KO reduces ARVCF protein expression in lymphoma cell lines. (c) VIM-KO upregulates the transcription of the short 3⁣′UTR isoform of *RRAGA*. (d) VIM-KO downregulates the transcription of the long 3⁣′UTR isoform of *RRAGA*.

The downregulation of ARVCF, a protein associated with APA, has been implicated in tumor progression. To further elucidate the mechanisms by which VIM-KO promotes lymphoma cell activity, we investigated the impact of VIM-KO on ARVCF expression. Western blot analysis revealed a reduction in ARVCF protein levels in VIM-KO lymphoma cells, including both A20 and M12 cell lines (Figure 5a,b).

Concomitant with the downregulation of ARVCF, the transcription of RRAGA, a key regulator of mTOR signaling, exhibited a shift in APA patterns. Specifically, the transcription of the short 3⁣′UTR isoform of RRAGA was upregulated, while the long 3⁣′UTR isoform was downregulated (Figure 5c,d).

**Figure 6:**
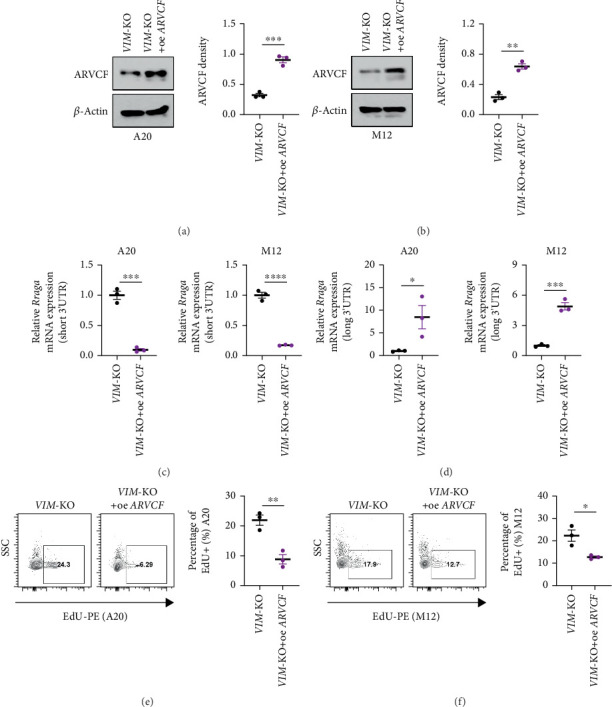
ARVCF overexpression restores RRAGA 3⁣′UTR length and inhibits lymphoma cell proliferation. (a, b) Western blot analysis confirms successful ARVCF overexpression in VIM-KO lymphoma cell lines. (c) ARVCF overexpression suppresses transcription of the short 3⁣′UTR isoform of *RRAGA*. (d) ARVCF overexpression restores transcription of the long 3⁣′UTR isoform of *RRAGA*. (e, f) ARVCF overexpression inhibits the proliferative capacity of lymphoma cell lines.

To understand the biological significance of altered ARVCF expression in lymphoma cells, we overexpressed ARVCF in VIM-KO cells using a lentiviral vector. Western blot analysis confirmed efficient ARVCF overexpression in both A20 and M12 cell lines (Figure 6a,b). Mechanistically, ARVCF overexpression in VIM-KO cells reduced the transcription of the short 3⁣′UTR isoform of RRAGA and increased the transcription of the long 3⁣′UTR isoform, thereby restoring RRAGA's normal APA patterns (Figure 6c,d). Importantly, restoration of the long 3⁣′UTR isoform transcription reversed the enhanced proliferative capacity of VIM-KO lymphoma cells (Figure 6e,f).

**Figure 7:**
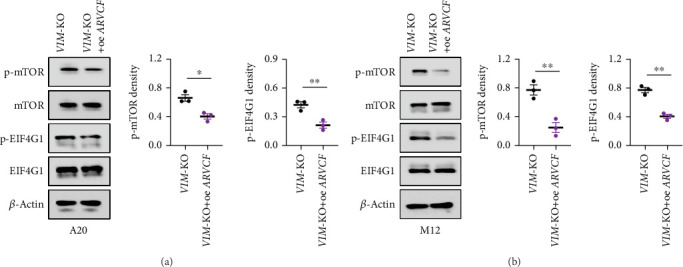
ARVCF overexpression suppresses mTOR-EIF4G1 signaling pathway activity. (a, b) ARVCF overexpression attenuates the VIM-KO–induced upregulation of mTOR-EIF4G1 signaling activity in lymphoma cell lines.

Finally, we assessed the effect of ARVCF overexpression on the activity of the mTOR-EIF4G1 signaling pathway in VIM-KO cells. ARVCF overexpression attenuated the hyperactivation of the mTOR-EIF4G1 pathway induced by VIM-KO, as evidenced by decreased phosphorylation levels of mTOR and EIF4G1 (Figure 7a,b). These findings suggest that VIM-KO promotes lymphoma progression by downregulating ARVCF, which in turn disrupts RRAGA APA patterns, leading to overactivation of the mTOR-EIF4G1 pathway.

## 3. Method

### 3.1. RNA-seq Experiments

Total RNA was isolated using TRIzol reagent (Invitrogen), with quality confirmed by NanoDrop. Libraries were prepared from poly-A-enriched mRNA through fragmentation, double-stranded cDNA synthesis, end-repair/adenylation, adapter ligation (Illumina), and size selection (400–500 bp, AMPure XP). After quantification (Bioanalyzer 2100), paired-end sequencing (150 bp) was performed on NovaSeq X Plus. Raw reads were quality-filtered (Cutadapt v1.15; Phred score ≥ 20), aligned to the reference genome (Genus species, Assembly ID) using HISAT2 (v2.0.5), and quantified via HTSeq (v0.9.1). Differential expression analysis (|log_2_FC| > 1, adj. *p* < 0.05, DESeq2 v1.30.0) was followed by functional enrichment (GO: topGO; KEGG: clusterProfiler v3.4.4; *p* < 0.05), with hierarchical clustering of DEGs (pheatmap, Euclidean distance).

### 3.2. RNA-seq Data Processing

The raw sequencing data underwent quality control assessments using FastQC (v0.12.1). Samples that did not meet quality standards due to issues such as poor base quality scores or high duplication levels were resequenced. Trimmomatic (v0.39) was utilized to trim sequences affected by adapter contamination and low-quality reads. The resulting trimmed FASTQ files were aligned to the mm10 mouse genome reference using STAR (v2.7.11). Expression levels of annotated transcripts were quantified using Htseq-count (v2.0.1) on the aligned BAM files. Differential gene expression analysis was performed with the R package DESeq2 (v3.21).

For the identification of APA events between sample groups, Bedtools (v2.31.0) genomecov was applied to the aligned BAM files to generate BedGraph files. These files were subsequently analyzed using DaPars to identify proximal polyadenylation sites and detect significant APA events. PDUI (percentage of distal polyA site usage index), as the primary output metric of DaPars, quantitatively measures the relative preference for distal polyA site utilization in transcript isoforms, where expression values are derived from RNA-seq coverage of polyA site regions. PDUI ranges from 0 to 1, with higher values indicating preferential usage of distal polyA site.

### 3.3. Functional Enrichment Analysis

To assess the biological functions of the DEGs and APA events, GO analysis and KEGG pathway enrichment analysis were performed using the R package clusterProfiler (v3.0.4). Additionally, pathway enrichment was further explored using the web-based tool Enrichr (https://maayanlab.cloud/Enrichr/enrich#).

### 3.4. Protein Experiments

#### 3.4.1. Protein Extraction and Digestion

Samples were processed with buffer-specific protocol protein solutions and plant tissues in SDT buffer (4% SDS, 100 mM Tris-HCl, pH 7.6) and cells and animal tissues in SDC buffer (5% SDC, 100 mM Tris-HCl, pH 8.5). Mechanical homogenization was applied to solid samples, while serum/plasma was directly centrifuged (14,000 × *g*, 20 min). Clarified lysates were quantified by BCA assay, and 15 *μ*g protein per sample underwent SDS-PAGE separation (4%–20% gel, 180 V, 45 min) followed by Coomassie staining. Gel segments were reduced with 10 mM DTT (37°C, 1.5 h), alkylated with IAA (room temperature, dark, 30 min), and digested with trypsin (1:50 *w*/*w*, 37°C, 15–18 h). Peptides were desalted (MCX column), vacuum-concentrated, and reconstituted in 0.1% formic acid.

#### 3.4.2. LC-MS/MS Analysis

Peptides were analyzed using an Orbitrap Astral mass spectrometer coupled to a Vanquish Neo UHPLC system in data-independent acquisition (DIA) mode. Full scans (380–980 m/z) used 240,000 resolution at 200 m/z. MS2 acquisition employed 299 variable isolation windows (2 m/z width) with HCD fragmentation at 25 eV collision energy.

#### 3.4.3. Data Processing

DIA-NN software (Version 1.8.1) performed library-free analysis using tryptic digestion rules (maximum 1 missed cleavage), fixed carbamidomethyl modification (cysteine), variable modifications (methionine oxidation and N-terminal acetylation). Protein identification confidence was set at false discovery rate ≤ 1%.

### 3.5. Protein Data Analysis

Hierarchical clustering was performed using Cluster 3.0 and Java Treeview with Euclidean distance and average linkage. Subcellular localization was predicted via CELLO's multiclass SVM system. Protein domains were annotated using InterProScan against the Pfam database. GO annotation was conducted by BLAST+ and InterProScan followed by Blast2GO mapping; KEGG pathway annotation utilized the KEGG database. Enrichment analysis employed Fisher's exact test with Benjamini–Hochberg correction (FDR < 0.05). GSEA was implemented in R (clusterProfiler v4.4.4) using fold change sorted protein expression matrices. Protein–protein interaction networks were constructed with STRING and visualized in Cytoscape (v3.2.1), with node centrality assessed by degree calculation. Principal component analysis (PCA) was performed in SIMCA-P (v14.1) after Pareto scaling. Coexpression networks were generated via WGCNA (R v1.69) using log2-transformed abundance data. Transcription factors and kinases were annotated using species-specific databases (PlantTFDB, AnimalTFDB, PhosphoSitePlus/iTAK/Phospho.ELM).

### 3.6. Cell Culture

The A20 and M12 cell lines were obtained from Cyagen Biosciences (Guangzhou, China). Cells were cultured in complete DMEM supplemented with 10% fetal bovine serum (FBS) (#10099141C, Invitrogen, United States) and a penicillin–streptomycin–glutamine mixture (#10378016, Invitrogen, United States) at 37°C in a humidified atmosphere containing 5% CO_2_. The culture medium was refreshed every 3 days.

### 3.7. Lentiviral Infection

Lentiviral vectors for ARVCF overexpression and corresponding controls were purchased from GenePharma (China). Lymphoma cell lines were infected with lentivirus in the presence of polybrene (#BL628A, Biosharp, China) for 12 h. After 48 h, infected A20 and M12 cells were subjected to antibiotic selection.

### 3.8. EdU Incorporation Assay

Cell proliferation was assessed using the BeyoClick EdU-594 assay kit (Beyotime, #C0078S). Log-phase cells were seeded in six-well plates (Corning, #3516) and incubated with 10 *μ*M EdU for 2 h. After incubation, the medium was discarded and cells were washed with PBS (#10010023, Gibco), fixed with 4% paraformaldehyde (Sigma, #158127) for 15 min, and permeabilized with 0.5% Triton X-100 (Sigma, #T8787) for 20 min. The Click reaction solution containing Alexa Fluor 488 azide was prepared according to the manufacturer's instructions and incubated with the cells in the dark for 30 min. After washing, nuclei were stained with PBS containing either 5 *μ*g/mL DAPI (Thermo Fisher, #D1306) or 7-AAD (BD Biosciences, #559925) for 15 min. Data were acquired using a flow cytometer (e.g., BD FACSCanto II).

### 3.9. CCK-8 Assay

Cells were seeded in 96-well plates at approximately 5 × 10^3^ cells per well, with three technical replicates per group. Blank controls (medium only) and negative controls (cells + medium) were also included. After 24, 48, or 72 h of incubation, 10 *μ*L of CCK-8 solution (Beyotime, #C0037) was added to each well, gently mixed, and incubated for 1–4 h (optimized by preliminary experiments). Absorbance at 450 nm was measured using a microplate reader (Thermo Fisher). Cell viability was calculated as follows:

 $$\begin{array}{c} \text{Cell viability }\left(\%\right)=\left(\text{O}{\text{D}}_{\text{exp}}-\text{O}{\text{D}}_{\text{blank}}\right)/\left(\text{O}{\text{D}}_{\text{control}}-\text{O}{\text{D}}_{\text{blank}}\right)\times 100\% \end{array}$$

All steps were performed under low-light conditions to avoid fluorescence interference, and care was taken to prevent bubbles from affecting the optical density readings.

### 3.10. Quantitative Real-Time PCR (qRT-PCR)

Total RNA was extracted using the RNA isolation kit (#R711-01, Vazyme), and cDNA was synthesized with the HiScript III RT SuperMix for qPCR (+gDNA wiper) (#R323-01, Vazyme) according to the manufacturer's instructions. qRT-PCR was performed using the MagicSYBR Mixture (#CW3008, CWBIO, China). For microRNA detection, the TaqMan MicroRNA Reverse Transcription Kit (#4366596, Invitrogen, United States) was used, and hsa-miR-615 was detected using a specific TaqMan probe (Assay ID: 001960, Applied Biosystems, United States). U6 snRNA (Assay ID: 001973) served as an internal control. Reactions were run on a Bio-Rad CFX96 Real-Time PCR Detection System (Bio-Rad, United States). All samples were analyzed in triplicate, and each experiment was independently repeated three times. Relative gene expression was calculated using the 2-*ΔΔ*Ct method.

### 3.11. Western Blot Analysis

Total protein was extracted using RIPA lysis buffer and quantified prior to electrophoresis. Equal amounts of protein were separated by 10% SDS-PAGE and transferred to 0.45-*μ*m PVDF membranes. Membranes were blocked with 5% nonfat milk for 2 h and incubated overnight at 4°C with the following primary antibodies: mTOR (66888-1-Ig, Proteintech, 1:1000); Phospho-mTOR (Ser2448) (67778-1-Ig, Proteintech, 1:1000); EIF4G1 (67199-1-Ig, Proteintech, 1:1000); Phospho-eIF4G (Ser1108) (2441, Cell Signaling Technology, 1:1000); ARVCF (4B1, sc-23874, Santa Cruz); *β*-actin (66009-1-Ig, Proteintech, 1:1000). After washing, membranes were incubated with HRP-conjugated secondary antibodies at room temperature for 1 h. Protein bands were visualized using an ECL detection kit (Beyotime, Shanghai) and imaged with the Tanon 4600 system (Tanon Science and Technology Co. Ltd.).

## 4. Discussion

This study reveals that VIM-KO in A20 B cell lymphoma cells induces widespread 3⁣′UTR shortening, with 4089 genes exhibiting global APA shifts. Mechanistically, VIM loss leads to downregulation of the APA regulatory protein ARVCF, which promotes APA shortening of RRAGA, a key activator of mTOR signaling. The resulting upregulation of RRAGA enhances mTOR pathway activity and supports increased lymphoma cell survival and proliferation. These findings establish a previously unrecognized VIM–ARVCF–RRAGA–mTOR regulatory axis that connects cytoskeletal integrity to posttranscriptional control and oncogenic signaling in lymphoma. Clinically, high VIM expression correlates with poor prognosis, particularly in ABC-subtype DLBCL, suggesting its utility as a biomarker for risk stratification. Therapeutic targeting of this axis—via VIM inhibition, mTOR blockade, or combination strategies—may overcome MDR and improve outcomes in refractory DLBCL [15].

APA is a crucial posttranscriptional mechanism that regulates mRNA stability, localization, and translation by modulating 3⁣′UTR length. In cancer, widespread 3⁣′UTR shortening enhances oncogene expression by eliminating regulatory elements such as microRNA binding sites [16, 17]. Large-scale analyses, including TCGA data, reveal recurrent tumor-specific APA changes that often go beyond simple shortening or lengthening patterns [17]. APA shortening is frequently linked to proliferative states and increased protein output [18]. Single-cell studies further show that APA alterations occur broadly across tumor and stromal cells, implicating APA in the global reprogramming of the tumor microenvironment [16]. In our study, VIM-KO lymphoma cells exhibit extensive APA shortening enriched in prosurvival pathways, including mTOR and G2-M checkpoint signaling. These findings underscore APA's role not only as a marker of proliferation but as an active contributor to oncogenic transcriptome remodeling in lymphoma.

Although ARVCF has been implicated in RNA processing through its interaction with splicing factors like hnRNPH, no direct evidence has been reported linking VIM or APA modulation to ARVCF, and our findings suggest a novel regulatory axis in which ARVCF may serve as a critical mediator. Proteomic analysis revealed that VIM deficiency in A20 cells leads to a marked downregulation of ARVCF, a member of the p120 catenin family involved in cell adhesion and signal transduction [19]. While traditionally studied for its role in cytoskeletal dynamics and cell–cell junctions, emerging evidence implicates ARVCF in malignancies such as non–small cell lung cancer, where its overexpression correlates with enhanced proliferation, invasion, and poor prognosis [15]. Notably, ARVCF has also been shown to interact with nuclear splicing factors and regulate alternative splicing [14, 20], raising the possibility that it may influence APA machinery more broadly. We hypothesize that VIM loss compromises ARVCF expression, thereby indirectly promoting APA shortening events such as that observed in RRAGA.

The RagA GTPase, encoded by the RRAGA gene, plays a crucial role in the mTORC1 pathway by facilitating the recruitment of mTORC1 to the lysosomal surface in response to amino acid signals [21–23]. In lymphoma cells, this nutrient-sensing pathway may also play an important role in controlling cell growth, survival, and response to stress. Our study reveals that the loss of VIM results in a significant shift in the posttranscriptional regulation of RRAGA, marked by global APA shortening. This alteration may enhance the stability and translation of RRAGA mRNA, thereby increasing the expression of RagA and promoting mTOR activation. The mTOR pathway is a well-known driver of oncogenic signaling, particularly in cancer cells, where its activation supports enhanced proliferation and resistance to apoptosis [24, 25]. Importantly, the VIM–ARVCF–RRAGA–mTOR regulatory axis connects the structural integrity of the cytoskeleton to the modulation of posttranscriptional events, facilitating mTORC1-driven oncogenesis in lymphoma.

This study has several limitations. First, while we establish the VIM–ARVCF–RRAGA–mTOR axis in lymphoma, the precise mechanistic details of VIM's impact on ARVCF and APA regulation remain unclear. Future research should employ genetic and pharmacological approaches to further explore these interactions. Second, our findings are based on A20 lymphoma cell lines and thus may not fully capture the APA heterogeneity across lymphoma subtypes. For example, BL exhibits APA shortening at proliferation genes, while DLBCL shows preferential 3⁣′UTR alterations in immune-evasion pathways. So, validation in other lymphoma subtypes or patient samples is necessary. Additionally, while APA shortening enhances oncogene stability, the broader effects on protein expression and cellular behavior require further investigation using high-throughput proteomic and RNA-seq methods. Finally, the therapeutic potential of targeting the VIM–ARVCF–RRAGA–mTOR axis needs to be explored through preclinical and clinical studies.

This study identifies the VIM–ARVCF–RRAGA–mTOR regulatory axis as a critical link between cytoskeletal integrity and oncogenic signaling in lymphoma. VIM loss leads to APA shortening, upregulating RRAGA and enhancing mTOR activity, promoting lymphoma cell survival and proliferation. Future studies are needed to further elucidate the mechanisms of this axis and assess its clinical relevance in lymphoma treatment. Among them, it is critical for providing evidence in vivo validation to recapitulate the tumor microenvironment that cannot be modeled in cell systems, assess the therapeutic potential of targeting this axis in a physiologically relevant context, and evaluate potential compensatory mechanisms that may emerge in whole-organism settings.

## Data Availability

The datasets generated and/or analyzed during the current study are available from the corresponding authors upon reasonable request.
