# Correction to “Vimentin Regulates Alternative Polyadenylation and mTOR Signaling via ARVCF to Promote B Cell Lymphoma Progression”

**DOI:** 10.1155/humu/9814094

**Published:** 2026-07-21

Shao, Lujing, Xing, Qianke, Xiong, Yao, Jin, Kaidi, Li, Qi, Dong, Chunyan, Ye, Qianling, Vimentin Regulates Alternative Polyadenylation and mTOR Signaling via ARVCF to Promote B Cell Lymphoma Progression, Human Mutation, 2025, 1463685, 15 pages, 2025. https://doi.org/10.1155/humu/1463685

Funding part: “The study is supported by the Science and Technology Development Fund of Pudong New Area (PKJ2022‐Y19) and the Young Medical Talents Training Program of Pudong Health Bureau of Shanghai (PKJ2023Y36)” was incorrect. This should have read “The study is supported by the Young Medical Talents Training Program of Pudong Health Bureau of Shanghai (PWRq2022‐01) and Science and Technology Development Fund of Pudong New Area (PKJ2023‐Y36).”.

We apologize for this error.

